# Comparative Study of Durability Behaviors of Thermoplastic Polypropylene and Thermosetting Epoxy Exposed to Elevated Temperature, Water Immersion and Sustained Bending Loading

**DOI:** 10.3390/polym14142953

**Published:** 2022-07-21

**Authors:** Ping Zhou, Jingwei Tian, Chenggao Li, Zhecheng Tang

**Affiliations:** 1 Key Laboratory of Structures Dynamic Behavior and Control, Ministry of Education, Harbin Institute of Technology, Harbin 150090, China; pingzhou2021@126.com (P.Z.); hittianjingwei@163.com (J.T.); zhechengtang@126.com (Z.T.); 2 Key Laboratory of Smart Prevention and Mitigation of Civil Engineering Disasters, Ministry of Industry and Information Technology, Harbin Institute of Technology, Harbin 150090, China; 3 School of Civil Engineering, Harbin Institute of Technology, Harbin 150090, China

**Keywords:** thermoplastic polypropylene, thermosetting epoxy, water immersion, sustained bending loading, mechanical properties, degradation mechanism, long-term life prediction

## Abstract

The long-term degradation of epoxy as the matrix and adhesive serviced in harsh environments plays a key role in engineering applications. Understanding how to improve the toughness and durability of epoxy through reasonable material replacement and design is significant to prolong the service life of engineering structures. In the present paper, thermoplastic polypropylene and thermosetting epoxy were exposed in a coupling environment of elevated temperature, water immersion and sustained bending loading. The evolutions of mechanical and thermal properties were further analyzed and compared. Long-term life prediction was conducted to evaluate the corrosive resistances of polypropylene and epoxy. It can be found that polypropylene has better hydrophobic behavior compared to epoxy. At 80 °C, the ratios of the diffusion coefficient and saturated water uptake between the two matrices were 114.4 and 2.94. At the longest immersion time of 90 days, the degradation percentages of tensile strength were 4.7% (40 °C), 7.5% (60 °C) and 8.8% (80 °C), respectively, which had the higher strength retention (>90%). The maximum strength increase in the multiples of polypropylene/epoxy and polypropylene/polyurethane was 1.95 and 1.75, respectively. The bending loading led to a maximum increase in tensile strength (~1.47%) owing to the oxygen isolation effect. The degradation mechanism was attributed to the active functional groups from the production process reacting with oxygen, resulting in the fracture of the local chain segment. By comparison, water molecules reacted with the hydroxyl groups or interrupted the intermolecular Van der Waals force/hydrogen bond of the epoxy, resulting in irreversible hydrolysis and property degradation. Through the comparison, it can be found that polypropylene and its composites have outstanding properties compared to epoxy, which can make them achieve great application prospects in engineering applications when considering a complex service environment.

## 1. Introduction

Polymers have been widely used as coatings, fiber reinforced polymer (FRP) composite and adhesives to reinforce, strengthen and repair newly built structures. For the coating, the main types are fire-proof coating, corrosion-resistant coating and friction-resistant coating [1,2,3,4], such as epoxy nanocomposites with some special functions [5,6]. For the resin matrix, its function is to fix and protect the fibers in composites and achieve superior mechanical properties [7,8]. For the adhesive, it is mainly used to strengthen and repair the structures through the interface bonding between the base material (such as steel and concrete components) and reinforcement materials (such as FRP) [9,10,11,12]. The polymer with high toughness, strength, durability and fatigue resistance is significant for material design and engineering application [13].

Thermosetting and thermoplastic matrices are two main categories of polymers. Generally, thermosetting matrix (such as epoxy and polyurethane) can be used as the matrix of FRP [14], which has the advantages, such as simple processing technology and production efficiency, high strength and lower viscosity [15], which lead to a much wider use of epoxy resin composites. However, the three-dimensional network cross-linking structures of epoxy lead to poor fracture toughness, which is the key factor in brittle failure and fatigue damage for FRP [16,17]. In addition, the molecular structure of epoxy contains a large number of hydroxyl groups, which easily form hydrogen bonds with water molecules in a service environment, resulting in the hydrolysis and plasticization of the matrix and fiber/matrix interface debonding [18,19]. In addition, epoxy cannot be melted and recycled after the curing, which will pollute the environment and causes a waste of resources. For the complex service environment (such as UV irradiation, elevated temperatures, moisture, corrosive medium and static/dynamic loading) and long life needs of civil structures (generally more than 50 years), the epoxy matrix is prone to fatigue damage due to insufficient toughness, resulting in the infiltration of corrosive solution and stress concentration, which is brought about the mechanical and chemical degradations of matrix and FRP [20,21,22]. Related research indicated that the water molecule entered the epoxy and caused irreversible hydrolysis and recovered plasticization [23,24,25]. Furthermore, the degradation of mechanical properties was controlled by the molecular chain breakage and microcracks from the hydrolysis [26,27]. E. Pe’rez-Pacheco et al. [28] found that the degradation mechanism of matrix failure mechanisms exposed to humidity conditions was attributed to plasticization, with swelling stresses due to hydrolysis. Furthermore, the plasticization of the epoxy matrix by moisture led to a decrease in the glass-transition temperature (Tg) and mechanical properties of the composite. For example, the tensile strength of composite laminates showed a decrease but after a moisture uptake of approximately 0.45%, it remained constant. The temperature dependence of the equilibrium water uptake of carbon fiber reinforced epoxy composites was quantitatively determined by Suh et al. [29] by assuming two different physical states of bound water and free water. The effect of temperature dependence on bound water was described by a negative value of heat of sorption in Henry’s law. Concurrently, the free water was considered to be immobilized by irreversible microcavitational damage or crazing in hygrothermal processes. The above analysis is important to reveal the effect of water types on the performance degradation of epoxy and its composites. Wang et al. [30] found the micropore and free volume space in epoxy gradually increased after the immersion. The tensile strength decreased to the lowest point after the immersion in water and alkali for one month, and the decrease percentages at 20 °C and 60 °C water or 60 °C alkali solution were 24%, 28% and 22%, respectively. Hong et al. [31,32] found polyurethane has a higher water uptake than epoxy due to more hydrophilic polar groups. Furthermore, the tensile strength of the polyurethane decreased rapidly after the first month of immersion and then leveled off. This was attributed to the plasticization due to water ingress and the increase in crosslinking degree due to post-curing. In addition, carbon fiber/polyurethane interface bonding was found to be superior to the carbon fiber/epoxy, leading to higher retention of tensile properties. To sum up, epoxy and polyurethane were sensitive to hygrothermal immersion [33]. For example, Pomies et al. [34] adopted the micromechanical finite element analysis to analyze the tensile properties of dry and wet glass/epoxy, carbon/epoxy and glass/polyphenylsulfide composites loaded in transverse tension. They concluded that the transverse stress levels corresponding to the debonding initiation and matrix failure decreased with the water absorption, which had good agreement with the experimental observation. Furthermore, the effect of moisture absorption on the interfacial strength of polymeric matrix composites was investigated by Bradley et al. [35]. The matrices in the composites have been found to saturate in moisture absorption at a level of 0.6–2% of the weight when soaked at room temperature. The degradation of interfacial shear strength due to moisture absorption has been found to vary from 0–22% for different composites. They concluded the interfacial shear strength degradation with moisture absorption was associated with a decrease in interfacial strength rather than the matrix mechanical properties. In addition, Sideridis et al. [36] obtained the interlaminar shear strength of cross-ply glass-epoxy resin composites for seven different fiber directions with short-beam three-point-bending tests, before and after moisture conditioning. It was found that moisture absorption reduced ILSS and stiffness of the examined composites, leading to larger failure deflections. Concurrently, the direction of fibers strongly affected the load-deflection response of the composite. Li and Xian et al. [37,38,39,40,41,42] systematically studied the long-term durability of carbon/glass fiber hybrid composites exposed to different environments and loading. They found that the exposure temperatures accelerated the water absorption and diffusion in the hybrid composite owing to the filling effect of initial defects by water molecules. Furthermore, the diffusion of water molecules caused the hydrolysis and plasticizing of resin. Plenty of bound water formed between the epoxy and water molecule and weakened the Van der Waals force and hydrogen bond between the fiber and resin, finally leading to debonding of the fiber/resin interface, especially for the higher exposed temperature and loading. This further contributed to the degradation of mechanical and thermal properties of composites. 

Compared to thermosetting epoxy, the thermoplastic matrix has a more optimal elongation at breaks, fracture toughness, thermal resistance and recyclability [43,44]. Generally, the molecular chain of thermoplastic matrix is linear or branched, and no chemical bonds form between the molecular chains [45]. It can be recycled through a reversible physical variation, such as heating melting and cooling molding. The above process had little influence on mechanical properties and microstructures [46]. Owing to its high viscosity and low strength, it is difficult to work with the fiber when considering the stress transfer between fiber and resin. When the viscosity of the thermoplastic resin is lowered and the fiber/resin interface bonding is improved, it can be expected that the thermoplastic matrix is more suitable for the complex service environment requirement. The common thermoplastic matrices mainly include polyethylene (PE), polyester (PET), polypropylene (PP), polyamide (PA), polycarbonate (PC) and polyether ether ketone (PEEK). Among them, polypropylene has advantages, such as rich raw materials source, low cost, easy processing and molding, better mechanical and thermal resistance, etc. Combined with the mature production technology, it has become the fastest growing synthetic matrix, which has the huge application potential to meet the need of FRP. When considering the complex environments (temperature/humidity, corrosive environment and dynamic/static loading) in civil engineering, the long-term service may lead to the degradation of mechanical properties of matrix and composite [47]. Therefore, it is necessary to study the mechanical property evolution of polypropylene exposed in coupling conditions of hygrothermal and loading. 

At present, the related investigation mainly focused on the long-term evolution of water absorption and mechanical properties of fiber-reinforced polypropylene composites. Arbelaiz et al. [48] investigated the effect of fiber/matrix modification and fiber content on water uptake and mechanical properties. The results revealed the mechanical properties of composites improved through adopting the maleic anhydride-polypropylene copolymer as a coupling agent and the water uptake rate clearly decreased. After the long water immersion, the mechanical properties drastically decreased. Panthapulakkal et al. [49] analyzed the mechanical, thermal and water uptake properties of hemp/glass fiber hybrid reinforced polypropylene composites. They found that hybridization with glass fiber can enhance the flexural properties and impact strength of the composite. However, prolonging the immersion time led to the decrease in the strength and modulus. Thermal properties and water absorption resistances of the hemp fiber composite were improved through the hybridization effect with glass fibers. Deng et al. [50] investigated the effects of water uptake on mechanical properties for woven fabric, glass fiber and natural fiber reinforced polypropylene composites. It can be found that three composites showed significant water uptake owing to the presence of voids, which depended on the fabric architecture and consolidation level. However, their mechanical properties were found to be largely unaffected by water absorption. On the other hand, some existing research had only concentrated on the photothermal aging in the natural environment for polypropylene [51,52,53]. C-H bond of polypropylene molecular structure was easily affected by light and heat. The produced active free radicals resulted in the continuous degradation reaction. In addition, polypropylene was easy to generate the carbonyl groups during the preparation process, which was the initiator of the photooxidation reaction, making polypropylene vulnerable to photooxidation aging [54]. L.A. Wall and S.E. Bresler et al. [55] considered that all aging reactions of polypropylene belonged to random cracking reactions. In the aerobic environment, polypropylene was easily affected by heat and light, resulting in an oxidative decomposition reaction, including chain initiation, chain growth and chain termination. Fiebig et al. [56] investigated the aging behavior of polypropylene at room temperature and annealing. They found that the amorphous and/or mesomorphic regions had variation at room temperature, which led to an increase in density and modulus related to embrittlement. In comparison, higher temperatures brought about the relaxation band recrystallization processes, which had a positive effect on impact strength. So far, the research on polypropylene exposure in a typical civil service environment is limited. Furthermore, the effects of temperature, humidity, loading on water absorption and mechanical properties of polypropylene were not yet clear. 

Based on the above analysis, it is necessary to find a high-performance matrix with superior corrosion/fatigue resistances to replace epoxy for use in civil engineering when considering the complex service environment. Furthermore, the knowledge of the aging behavior of high-performance matrix (such as degradation mechanism and service life) is significant for the design parameters of engineering applications. In the present study, the typical service environment was applied to investigate the long-term evolution of polypropylene and epoxy. At the same time, the comparison of long-term performance between epoxy and polypropylene was also conducted. The immersion temperatures were 40 °C, 60 °C and 80 °C, and the distilled water was adopted as the exposure medium. To simulate the real service condition, a bending fixture was designed to apply the bending loading, and the maximum bending strain in the midspan was 0.54%. Water uptake, mechanical and thermal properties tests were conducted to obtain the long-term evolution. The degradation mechanism of mechanical properties was revealed based on the microstructure analysis. Based on the Arrhenius theory, the long-term life predictions of mechanical properties were conducted. Finally, the application prospect analysis of polypropylene in structural engineering was analyzed and compared with epoxy and polyurethane in terms of failure strain, long-term property and fatigue resistance.

## 2. Materials and Methods 

### 2.1. Raw Materials and Sample Preparation

Thermoplastic polypropylene (PP) and thermosetting epoxy (EP) were adopted to conduct the water uptake, mechanical and thermal tests. For polypropylene, its type was isotactic polypropylene with a molecular weight of ~250,000, which was produced by Taixinlong Plastic Products Co., Ltd. (Dongguan, China) with a nominal rectangle sheet size of 300 mm × 300 mm × 2 mm. The surface of the matrix sheet was milky white with a density of 0.906 g/cm^3^. Bisphenol A epoxy from Dagong Composite Company (Linyi, China) was adopted and anhydride was used as a curing agent. The mass ratio of the above two components was 1:0.345. The molecular structure formula of epoxy can be found in the research [57] with the epoxy value of 0.51 and epoxy equivalent of 196. Furthermore, its density and glass transition temperatures (Tg) were 1.20 g/cm^3^ and 89.8 °C, respectively. The preparation process of the epoxy sheet was as follows. Firstly, the curing agent was added to the epoxy and then evenly stirred for 10 min, followed by oscillation with ultrasonic waves for 15 min to remove the bubbles. Then, the mixed solution was poured into an aluminum mold and cured at 60 °C for 24 h in an oven. After the curing, the resin sheets were cut into different sizes and polished with a polishing machine according to the experimental tests. 

### 2.2. Immersion Conditions

**Figure 1:**
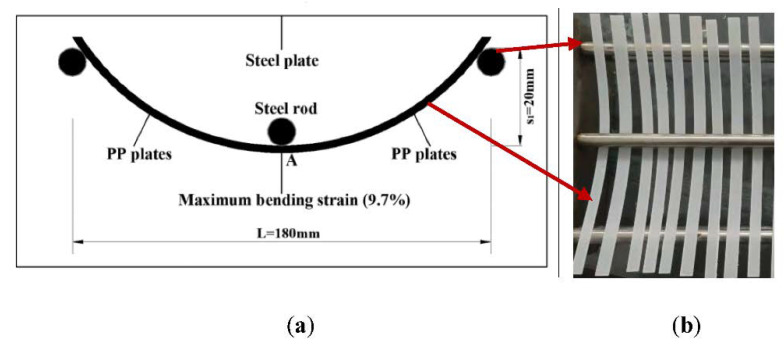
Schematic diagram and experimental photographs of bending device of polypropylene plate. (**a**) Schematic diagram; (**b**) Experimental photographs.

In order to realize the coupling exposure of loading and water immersion, a bending fixture was designed to apply the bending loading and its schematic diagram was as shown in Figure 1. Based on the plane strain theory, the maximum bending strain of matrix plate midspan was determined as follows [58]:

(1) $$\varepsilon_{x\max}=\frac{4vh}{L^{2}}$$

where *v* is the maximum bending deflection, and it is the sum of midspan bending deflection (20 mm) and specimen thickness (*h* = 2.0 mm), *L* is the specimen span (180 mm). Therefore, the maximum bending strain of the matrix plate in the midspan was calculated to be 0.54%.

The exposure conditions of two kinds of matrices under the loading and unloading conditions were shown in Table 1. As shown, the exposure temperatures were 40, 60 and 80 °C and the exposure intervals were 0, 30, 60 and 90 days, respectively. It should be noted that the above exposure intervals were determined by referring to the others’ work [31,59]. Distilled water was adopted as the exposure medium. It was worth mentioning that the maximum bending strain (0.54%) was applied to the polypropylene plate only for the exposure temperature of 60 °C. This was to analyze the effect of bending loading on the long-term performance evolution of polypropylene plates at the same exposure temperature. 

### 2.3. Water Uptake Test

Two kinds of matrices were put into a temperature-constant water tank with the temperatures of 40, 60 and 80 °C to conduct the water absorption test. Each interval time of the water uptake test was set as 4 h, 12 h, 1 day, 3 days, 7 days, 14 days, 21 days, 30 days, 60 days and 90 days, respectively. For each time interval, the samples were taken out from the water tank and dried with absorbent paper. An electronic balance with an accuracy of 0.1 mg was adopted to obtain the weight. After the weighing, the samples were put back into the water tank for continuous immersion. In order to reduce the experimental error, the whole weighing process was controlled within 15 min. Five samples were weighed and the average value was obtained. The water absorption of two kinds of matrices was obtained according to the following equation [60]:

(2) $$W(\%)=\frac{W_{t}-W_{0}}{W_{0}}\times 100\%$$

where *W_t_* is the wet weight at time *t*, and *W*_0_ is the control weight before the immersion. 

### 2.4. Tensile Test 

The tensile test of the polypropylene plate was conducted with the universal tensile machine (DHY-10080, Shanghai, China) according to ASTM D638-2010. The samples with the dumbbell shape were prepared to conduct the tensile test. The crosshead displacement rate of the tensile machine grip was 1 mm/min. Three samples were tested to obtain the average. The calculation of tensile strength was performed as follows:

(3) $$\sigma =\frac{P}{bh}$$

where *P* is the tensile breaking load, *b* is the sample width, and *h* is the sample thickness.

### 2.5. Thermogravimetric Analysis Test (TGA)

Long-term thermal properties of polypropylene and epoxy before and after the immersions were evaluated by thermogravimetric analysis test (TGA). For polypropylene, the effects of immersion temperature (40, 60 and 80 °C), time (30 and 90 days) and bending loading (0 and 0.54%) were considered. For epoxy, the control and aged for three months at 60 °C were adopted as the compartive group. The mass variations of two matrices were obtained by a TGA analyzer (NETZSCH STA 449C, Germany) from room temperature to 600 °C at a heating rate of 10 °C/min in air. One sample was tested with the weight of ~10 mg in powder and 20 mL/min dry air flow was applied during the test. Mass loss curves at elevated temperatures were tracked to analyze the decomposition rate and temperature.

### 2.6. Scanning Electron Microscopy (SEM)

The surface morphology of polypropylene and epoxy before and after the immersions were observed through the Scanning Electron Microscope (SEM, VEGA3). Before the tests, the samples were vacuumed and sprayed with gold to increase the electrical conductivity. The test frequency, electric current and voltage amplitude were set as 1000 Hz, 0.7 A and 30 kV, respectively. It was noted that the samples before and after the tensile test were adopted to analyze the fracture failure mode.

## 3. Results and Discussion

### 3.1. Water Absorption and Diffusion Behavior

**Figure 2:**
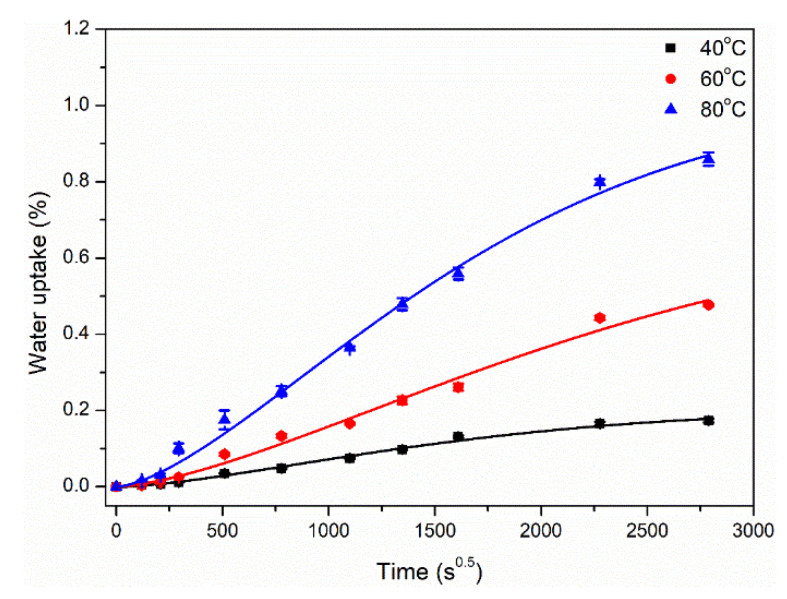
Water uptake curves of polypropylene immersed in distilled water at different temperatures. Note, solid lines represent the fitting curves using the Fick’s diffusion model.

Immersion in distilled water led to the weight gain of polypropylene and epoxy plates, and the water absorption curves of polypropylene plates for as long as 90 days were shown in Figure 2. It was noted that the water absorption data under the bending loading were not available. As shown, the water uptake curve of polypropylene presented a two-stage variation, including the initial fast increase and subsequent moderate increase. An increase in immersion temperature from 40 °C to 80 °C led to an increase in water uptake. For example, the maximum water uptakes at the longest immersion were 0.17% (40 °C), 0.48% (60 °C) and 0.86% (80 °C), respectively. This was because higher immersion temperature provided more energy for water molecules and accelerated their diffusion in the matrix. When water molecules were completely filled into the inner micropores of matrix, the water absorption saturation was realized. Therefore, the saturation time was shortened with the increase in immersion temperature. The water absorption process of polypropylene can be approximatively described through the simplified Fick’s diffusion model [61], as follows:

(4) $$M_{t}=M_{\infty }\left\{1-exp\left[-7.3{\left(\frac{Dt^{}{}_{}}{h^{2}}\right)}^{0.75}\right]\right\}$$

where *M_t_* is the water uptake at immersion time *t*, *M*_∞_ is the saturated water uptake, *D* is the diffusion coefficient, and *h* is the specimen thickness. Equation (4) and Origin 8.1 software were adopted to conduct the nonlinear fitting after assigning the initial values of *D* and *M*_∞_. By fitting, the dependences of water uptake on the immersion time at different temperatures were shown in Figure 2, and the water absorption fitting parameters were shown in Table 2. As shown, the saturated water uptake and diffusion coefficient of the matrix increased obviously with the immersion temperature. 

**Figure 3:**
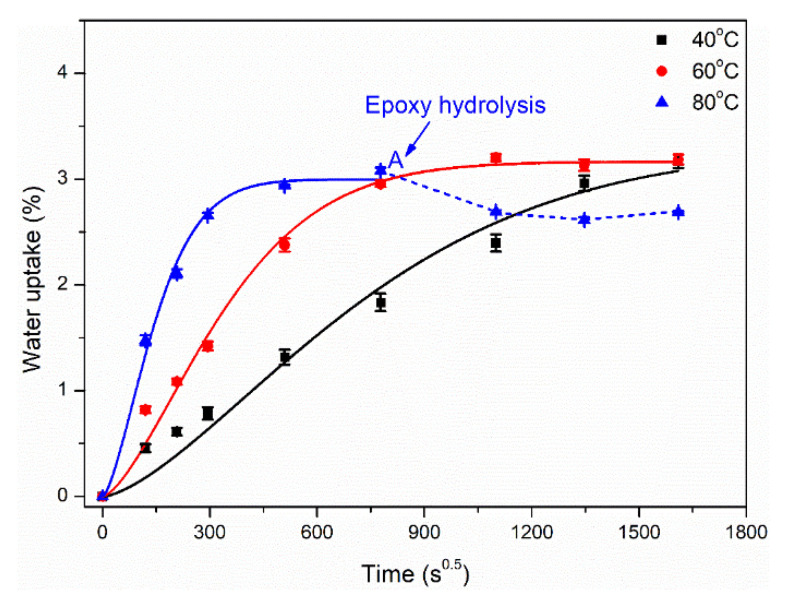
Water uptake curves of epoxy immersed in distilled water at different temperatures. Note, solid lines represent the fitting curves using the Fick’s diffusion model.

Similarly, the water uptake data and fitting curves of epoxy at different temperatures were shown in Figure 3. It can be seen that the higher immersion temperature (60 °C and 80 °C) accelerated the diffusion rate of water molecules, leading to a quickly saturated state. According to Fick’s model, the saturated water absorptions were obtained to be 3.30%, 3.16% and 3.00%, respectively. It was worth mentioning that a higher immersion temperature at 80 °C led to the epoxy hydrolysis after the saturated water absorption (point A). Furthermore, the water absorption curve decreased with the immersion time. This was attributed to the hydroxyl functional groups of the epoxy reacting with water molecules to form hydrogen bonds, which further interrupted the chain structure. In addition, the above hydrolysis process of the epoxy matrix was irreversible, which brought about the irreversible degradation of mechanical and thermal properties.

Water absorption fitting parameters of two kinds of matrices were presented in Table 2. By contrast, it can be found that the water absorption capacity of epoxy was far more than that of polypropylene in terms of saturated water uptake and diffusion coefficient.

**Figure 4:**
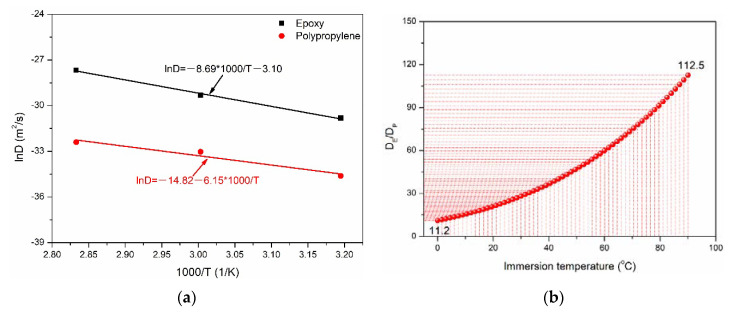
Diffusion coefficients of two kinds of matrices of (**a**) temperature dependence and (**b**) diffusion coefficient ratio.

Figure 4 quantitatively describes the dependence of diffusion coefficient on immersion temperature. As shown in Figure 4a, the diffusion coefficients of the two matrices were linear with the immersion temperature. However, the ln*D*-1000/*T* curve of epoxy was above that of polypropylene, indicating the faster diffusion behavior of water molecules in the matrix. For the same immersion temperature, the water absorption coefficient of epoxy was higher than that of polypropylene. Figure 4b shows the diffusion coefficient ratio of epoxy and polypropylene with the immersion temperature. Obviously, the diffusion coefficient of epoxy was significantly higher than that of polypropylene and increased with the immersion temperature. For example, when the immersion temperature was 90 °C, the maximum ratio of diffusion coefficient was 112.5. Therefore, it can be concluded that water molecules were easier to diffuse in epoxy than polypropylene at the same immersion temperature. Furthermore, higher water absorption and diffusion rates may accelerate the property degradation of epoxy.

**Figure 5:**
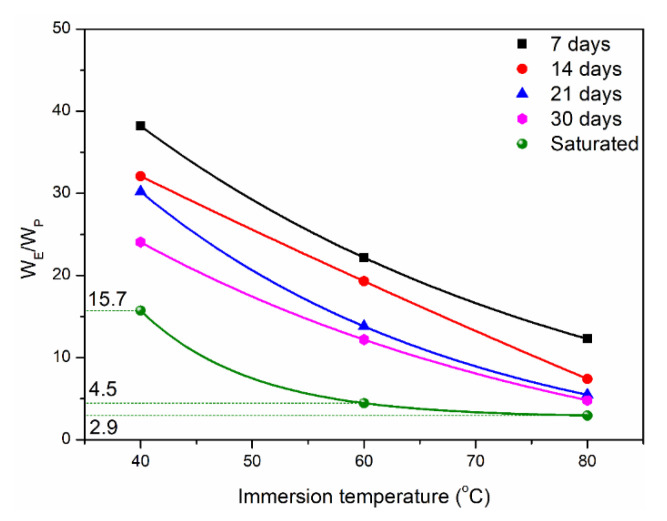
Water uptake ratio of two kinds of matrices at different immersion time.

Figure 5 shows the ratio of water absorption of two kinds of matrices at different immersion times. It can be found that the water absorption of epoxy was much higher than that of polypropylene. Furthermore, the maximum ratio of saturated water absorption between them was 15.7. Based on the above analysis, it can be seen that polypropylene has better hydrophobic behavior compared to epoxy owing to different molecular chain structures. The lower diffusion coefficient and saturated water absorption indicated the excellent durability of polypropylene, which will be verified in the following mechanical property’s evolution and life prediction. 

### 3.2. Effect of Immersion on Mechanical Properties

#### 3.2.1. Tensile Properties

**Figure 6:**
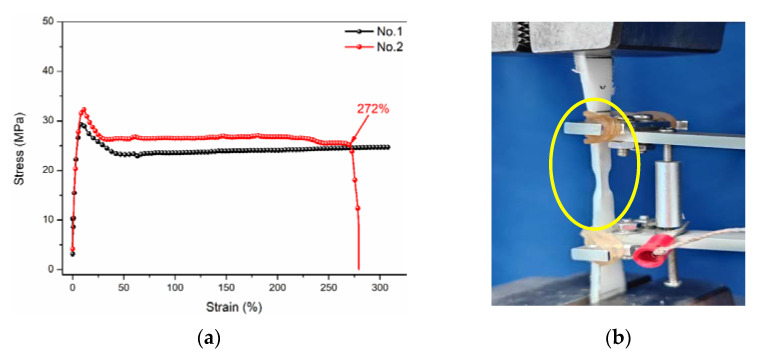
Tensile stress-strain curve (**a**) and tensile failure mode (**b**) of control polypropylene.

After analyzing the water absorption behavior, the effect of immersion on tensile properties was further investigated. Figure 6 shows the tensile properties of control polypropylene before the immersion, including the tensile stress-strain curve (Figure 6a) and tensile failure mode (Figure 6b). It can be found that the tensile stress-strain curve of polypropylene mainly included a three-stage variation. The first stage was a typical elastic deformation stage, and the stress strain presented the linear dependence. At the end of this stage, polypropylene achieved the ultimate bearing loading capacity. In the second stage, the matrix experienced a typical yield process, and the stress was gradually decreasing with the increase in strain. Finally, the third plastic deformation stage occurred. The strain was gradually increasing, while stress remained almost unchanged. At the end of this stage, polypropylene achieved the maximum tensile strain, and its value was 272% for the sample of No. 2. It can be further found that No. 1 did not have the final tensile failure. The difference in the elongation at break for the above two samples was acceptable within the error range. Figure 6b shows a typical failure mode of polypropylene during the tension. It can be seen that the obvious plastic deformation and shrinkage occurred (marked in yellow), which indicated the polypropylene had better toughness and higher fracture energy (the area enclosed by the stress-strain curve). When considering the actual engineering application, polypropylene can be designed according to the elastic stage, yield stage and plastic deformation stage to meet the material property requirement of the engineering application. 

**Figure 7:**
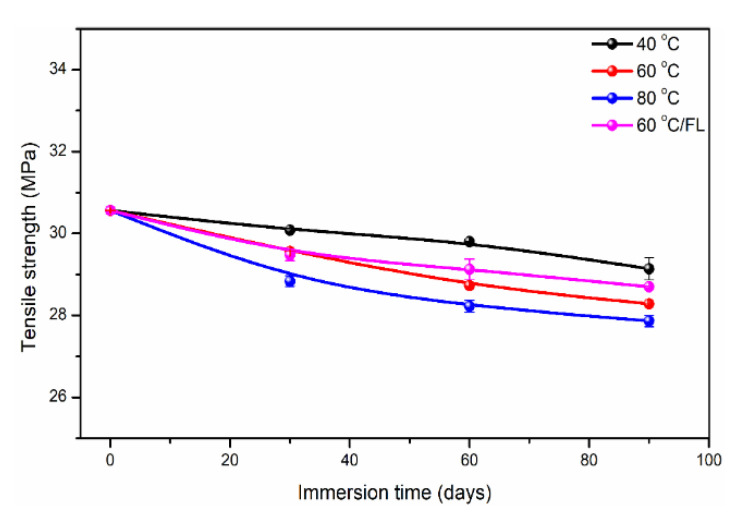
Tensile strength of polypropylene immersed at different temperatures and loadings. Note, FL is the flexural loading with the maximum flexural deflection of 20 mm.

After analyzing the tensile properties of the control sample, the effect of immersion on the tensile strength of polypropylene was shown in Figure 7. It was noted that the deformation of resin has not been monitored by the extensometer after the aging test. This was because the huge deformation of polypropylene during the tension may exceed the usage range of the extensometer. The sample of “0 days” was tested at room temperature to obtain the control tensile strength. As shown, the tensile strength of polypropylene decreased gradually with the increase in immersion temperature (40→80 °C) and time (0→90 days). At the longest immersion, the degradation percentages of tensile strength were 4.7% (40 °C), 7.5% (60 °C) and 8.8% (80 °C), respectively. For each immersion temperature (40, 60 and 80 °C), the tensile strength of polypropylene decreased with the increase in water absorption. The above degradation was possibly attributed to the following reason. Under the catalysis of external light, heat and oxidation [62,63], C-H bonds in the polypropylene chain segment were easy to break and produced more active free radicals, which caused the continuous degradation reaction. For the present exposure, high temperature and oxygen in distilled water acted as the catalysts of the matrix for chemical reactions. Furthermore, higher immersion temperatures accelerated the degradation process, resulting in a decrease in tensile strength. In addition, it can be found that the tensile strength retention of polypropylene under the bending loading was more than that of the no-loading condition. This was because the density of polypropylene was lower than that of water. During the process of immersion, the matrix always floated on the water without the bending loading, leading to direct contact with oxygen. Furthermore, the groups produced during the production processing reacted with oxygen, resulting in the fracture of local chain segments and a decrease in tensile strength [64]. In contrast, the bending loading immersed the polypropylene completely in water, reducing the direct contact with oxygen. Furthermore, the oxidation reaction was greatly weakened, resulting in higher tensile strength retention. Therefore, it can be seen that polypropylene was vulnerable to heat and oxygen during the immersion. Necessary isolation measures of external light, heat and oxidation should be adopted in practical engineering applications. 

#### 3.2.2. Comparison of Tensile Strength with the Epoxy and Polyurethane

**Figure 8:**
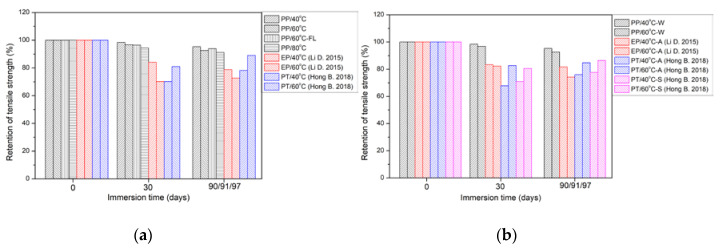
Comparison of tensile strength retention among polypropylene, epoxy and polyurethane of (**a**) distilled water environment and (**b**) alkali or salt solution. Note, PP, EP and PT denote polypropylene, epoxy and polyurethane, respectively. W, A and S represent the distilled water, alkali solution and salt solution, respectively [57,65].

The comparisons of tensile strength retention of polypropylene, epoxy and polyurethane immersed in distilled water, alkali or salt solution were conducted [57,65] and shown in Figure 8. It was noted that PP, EP and PT denote polypropylene, epoxy and polyurethane, respectively. For convenience of analysis, the strength degradation percentage of three resins was adopted. It was worth mentioning that polyurethane was selected for the comparison to analyze its corrosion resistance to distilled water, alkali and salt solutions. It can be found that polypropylene has high tensile strength retention (>90%) after the immersion for 90 days. In contrast, the retention of tensile strength of epoxy and polyurethane was basically 70~80%. In addition, the effects of alkali solution and salt solution on the tensile strength of the above two matrices were negligible compared to distilled water. From the perspective of molecular structure, polymers mainly rely on intermolecular chemical bonds, Van der Waals forces and hydrogen bonds to resist the external load [65]. The degradation mechanisms of tensile strength of three kinds of matrices were summarized as follows. For polypropylene, the groups from the production process reacted with oxygen, resulting in the fracture of local chain segments. Furthermore, higher exposure temperature and longer exposure time accelerated the above reaction, leading to an extra decrease in tensile strength. For epoxy, water molecules diffused into the matrix at elevated temperatures, and reacted with the hydroxyl groups of epoxy, resulting in reversible plasticization and irreversible hydrolysis (Figure 3), which contributed to the decrease in tensile strength [66]. For polyurethane, the degradation of tensile strength was attributed to the diffusion of water molecules in the matrix interrupted the intermolecular force between the chains, such as Van der Waals force and hydrogen bond [65]. To sum up, polypropylene was relatively sensitive to oxygen and the immersion in water brought about higher tensile strength retention. In comparison, epoxy and polyurethane were vulnerable to water immersion, and the breaking of molecular chain and the weakening of intermolecular force led to the degradation of tensile strength. Furthermore, the higher immersion temperature added the sensitivity of three kinds of matrices to the environment. 

After analyzing the degradation mechanism of tensile strength, the ratios of tensile strength retention of polypropylene compared to epoxy and polyurethane immersed in distilled water were obtained and shown in Table 3. As found, the maximum ratios of tensile strength were 1.95 (PP/EP) and 1.75 (PP/PT), respectively, which indicated polypropylene has better corrosion resistance compared with epoxy and polyurethane, which achieved great application potential when considering the harsh service environment.

### 3.3. Effect of Immersion on Thermal Properties

**Figure 9:**
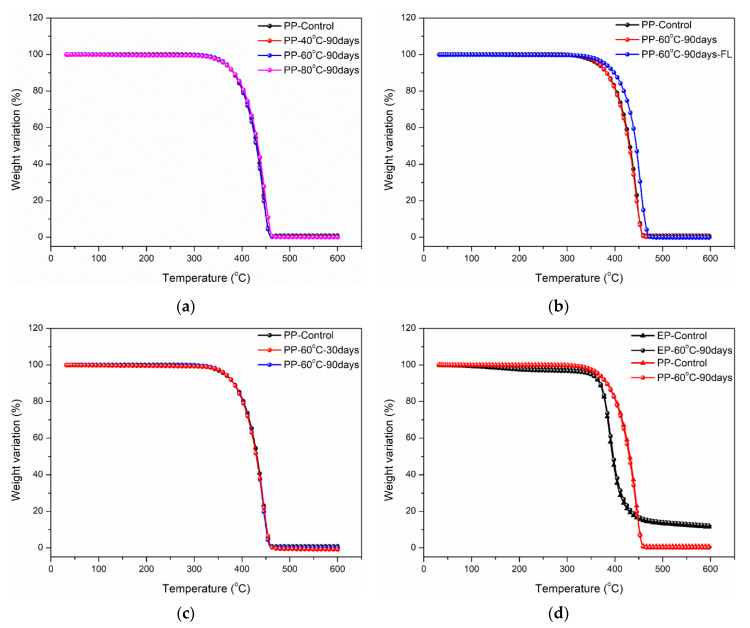
Effects of temperature, bending loading, immersion time and matrix type on weight variation of (**a**) temperature effect, (**b**) bending loading effect, (**c**) immersion time effect and (**d**) matrix type effect.

The effects of immersion in distilled water on the thermal properties of polypropylene and epoxy were analyzed through the weight variation curves at elevated temperatures as shown in Figure 9, including the temperature effect, bending loading effect, immersion time effect and matrix type effect. Furthermore, the maximum and ultimate decomposition temperatures were obtained from the maximum decomposition rate and inflection point of weight curves as shown in Table 4. As shown in Figure 9a,c, the effects of immersion temperature increase (40→80 °C) and time increase (30→90 days) on the weight variation of polypropylene were negligible, which contributed to the similar decomposition temperatures (Table 4). This is because polypropylene has a non-polar molecular structure, which is not sensitive to immersion temperature and time, indicating excellent thermal resistance after long-term immersion. In comparison, the bending loading led to a significant increase in weight retention at elevated temperatures (Figure 9b). This was because bending loading largely reduced the oxidation contact of polypropylene through completely immersing in water and improved its thermal resistance, which also contributed to a significant increase (~10 °C) in maximum and ultimate decomposition temperatures (Table 4). Figure 9d compares the weight variations of epoxy and polypropylene at elevated temperatures. It can be found that the weight variation curve of polypropylene was always above epoxy during the decomposition process. This was because the thermal decomposition temperature of epoxy resin decreased significantly due to hydrolysis and relaxation during long-term immersion. Furthermore, the effect of the above degradation on thermal performance is significant. To sum up, higher decomposition temperature and lower decomposition rate indicated polypropylene has better thermal resistance than epoxy before and after the immersions, which has good agreement with the evolution of mechanical properties. 

### 3.4. Surface Morphology Analysis 

**Figure 10:**
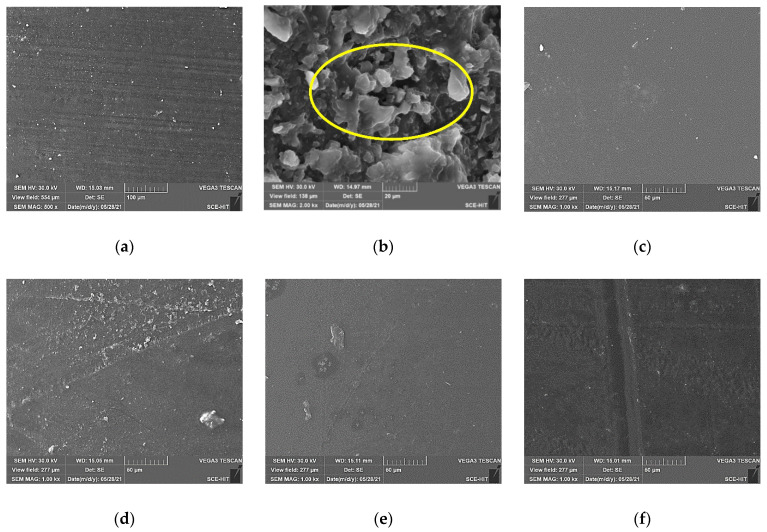
Surface morphology of two kinds of matrices before and after the immersions of (**a**) control-EP, (**b**) 60 °C-90-EP, (**c**) control-PP, (**d**) 60 °C-90-PP, (**e**) 60 °C-FL-90-PP and (**f**) 80 °C-90-PP.

The degradation mechanism of mechanical properties of polypropylene and epoxy after the immersion was revealed by surface morphology analysis as shown in Figure 10. For the control, it can be found that the surface of polypropylene was dense and smooth, while there were more microgrooves on the surface of the epoxy. After the immersion at 60 °C for 90 days, the surface of polypropylene had almost no change. However, the epoxy surface was obviously etched by the immersion, and the corrosive micropore and matrix peeling were observed, which verified the serious hydrolysis effect (Figure 3). When the immersion temperature increased to 80 °C, it can be found that the surface of polypropylene after the immersion became relatively rough and some small bulges were produced. In addition, the effect of bending loading on the surface morphology was not remarkable.

**Figure 11:**
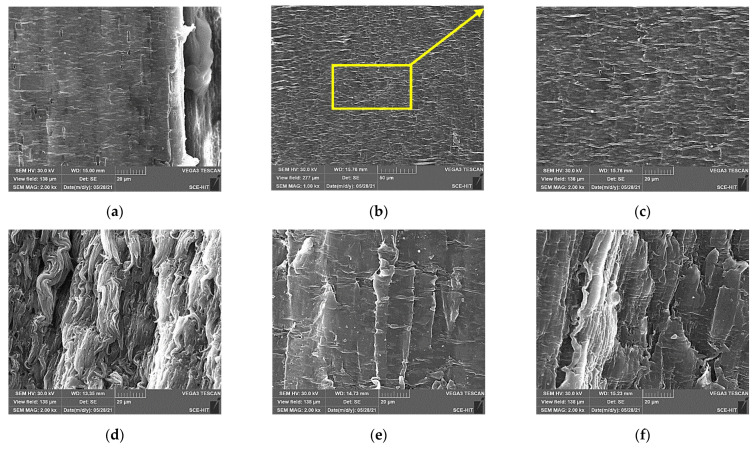
Surface fracture morphology of two kinds of matrices after the tensile tests of (**a**) control-EP, (**b**) control-PP (necking surface) (**c**) enlarged view of (**b**); (**d**) 60 °C-90-PP, (**e**) 60 °C-FL-90-PP and (**f**) 80 °C-90-PP.

The fracture surface morphology of two kinds of matrices after the tensile tests were shown in Figure 11. For the control (Figure 11a–c), it can be found that the obvious folds appeared on the surface of polypropylene owing to the tensile deformation. When the tension load was removed, the unrecoverable deformation produced the bulges and formed a fold effect, which indicated that polypropylene had good ductility and plastic deformation ability. From the necking surface, the micro-holes gradually appeared during the tension and the continuous expansion in the direction perpendicular to the tensile force contributed to the tensile fracture. After the immersion, it can be found that the immersion temperature had little effect on tensile fracture morphology (Figure 11a,d,f). Furthermore, the bending loading did not change the fracture failure mode. However, the long-term loading effect may weaken the toughness of polypropylene and further reduce the number of folds.

### 3.5. Long-Term Life Prediction of Tensile Strength

After analyzing the degradation mechanism, the long-term life predictions of tensile strength for polypropylene and epoxy were carried out to provide the durability design parameters for engineering applications. Arrhenius model was adopted for the single-factor dominant degradation mechanism during the immersion as follows [59]. Furthermore, the degradation rate of tensile strength was accelerated with the increase in immersion temperature.

**Figure 12:**
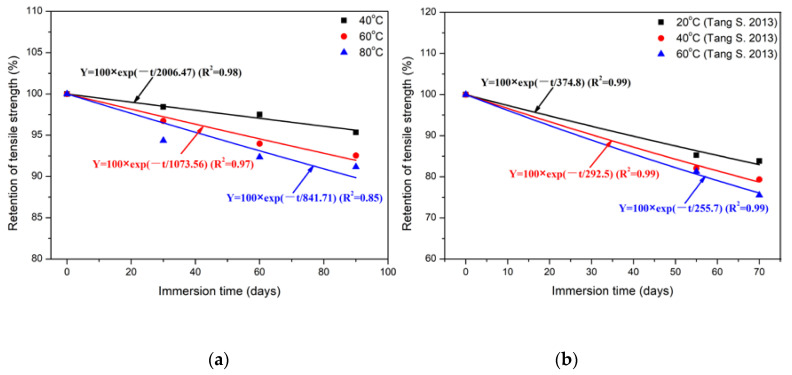
Fitted curves of tensile strength for two kinds of matrices using Equation (5) of (**a**) PP and (**b**) EP [67].

**Figure 13:**
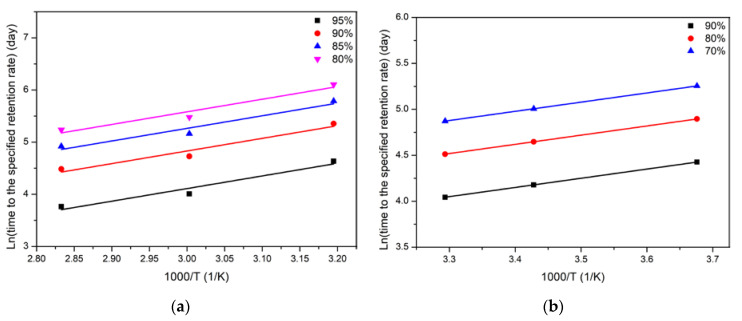
Arrhenius fitted curves of long-term life for tensile strength of two kinds of matrices (**a**) PP and (**b**) EP [61].

(5) Y=100exp(−t/τ)

where *Y* is the retention of tensile strength, *t* is the time and τ is the constant. It should be noted that the above model was adopted based on the following assumptions. For polypropylene, the increase in immersion temperature accelerated the oxidation reaction and led to a decrease in tensile strength. For epoxy, the increase in immersion temperature accelerated the diffusion of water molecules and hydrolysis or plasticization of epoxy, resulting in a decrease in tensile strength. Based on Equation (5), the fitted curves of tensile strength for two kinds of matrices were obtained and shown in Figure 12. It was noted that the data on the tensile strength of epoxy came from the research work of Tang et al. [67]. Furthermore, the regression coefficient (*τ*) and degree of fitting (*R*^2^) were shown in Table 5. It can be found that the tensile strength of two kinds of matrices decreased gradually with the increase in immersion temperature and time. Higher *R*^2^ implied that the Arrhenius model can well describe the tensile strength evolution. Furthermore, the degradation rate can be expressed through the Arrhenius relationship as follows [68]:

(6) $$\ln(\frac{1}{k})=\frac{E_{a}}{R}\frac{1}{T}-\ln A$$

where *E*_a_ is the activation energy of materials, *R* is the universal gas constant and *T* is the immersion temperature. Based on Equation (6), Arrhenius plots of tensile strength for two kinds of matrices were obtained and shown in Figure 13. As shown, the linear expression can be used to describe the relationship between the specific time for given strength retention and immersion temperature. Furthermore, the regression coefficient (*E*_a_/*R*) and degree of fitting (*R*^2^) of life prediction were shown in Table 6. It can be seen that the *R*^2^ were 0.91 (PP) and 0.99 (EP), respectively. 

According to Equation (6), the time-shift factor (TSF) for tensile strength to reach the same value at temperatures *t*_1_ and *t*_0_ can be calculated as follows.

(7) $$TSF=\frac{t_{0}}{t_{1}}=\frac{c/k_{0}}{c/k_{1}}=\frac{k_{1}}{k_{0}}=\frac{A\exp(-E_{a}/RT_{1})}{A\exp(-E_{a}/RT_{0})}=\exp(\frac{E_{a}}{R}(\frac{1}{T_{0}}-\frac{1}{T_{1}}))$$

Substituting the fitting parameters from Equation (6) into Equation (7), TSF can be obtained in three typical bridge service environments. It should be pointed out that polypropylene and epoxy were considered to be applied to bridge structures as the matrix and adhesive, etc. The above three typical bridge service environments included different geographical locations in China. Furthermore, the annual average temperatures of the above three bridge environments were 8.8 °C (Shenyang Youth Bridge), 15.9 °C (Jiangsu Yangtze River Bridge) and 26.9 °C (Hainan Century Bridge). Finally, the time-shift factor of life prediction of tensile strength for two kinds of matrices was obtained and shown in Table 7. 

**Figure 14:**
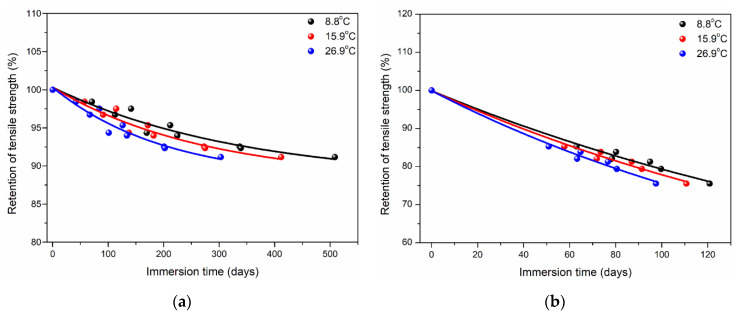
Long-term life predictions of tensile strength of two kinds of matrices (**a**) PP and (**b**) EP [61].

Based on Table 7, the long-term life predictions of tensile strength of two kinds of matrices were obtained and shown in Figure 14. As shown, the tensile strength of polypropylene decreased and tended to be stable with the increase in service time. In contrast, the degradation rate of tensile strength of epoxy was faster, leading to lower strength retention. For example, when the service time was 500 days, the retention of tensile strength for polypropylene was close to 90% at 8.8 °C. However, the retention of tensile strength of epoxy was only ~75% for the service time of 120 days. It can be seen that polypropylene has better corrosive resistance and service life than epoxy. 

### 3.6. Application Prospect Analysis of Polypropylene 

After obtaining the mechanical degradation and life prediction of polypropylene, its application prospect in structural engineering was also further analyzed through the comparison with epoxy and polyurethane. Comparative analysis among three kinds of matrices included failure strain, long-term property and fatigue resistance. 

#### 3.6.1. Failure Strain Analysis 

**Figure 15:**
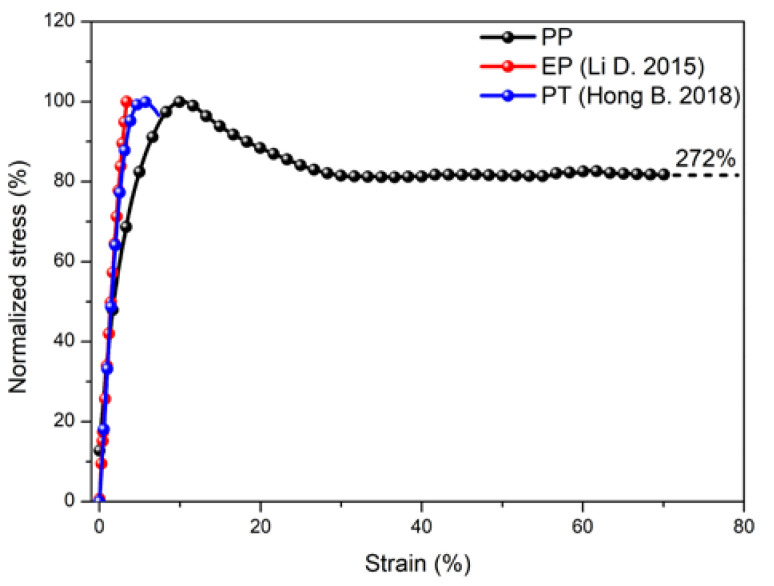
Comparison of tensile stress-strain curves of three kinds of matrices [57,65].

The tensile stress-strain curves of three kinds of matrices were compared and shown in Figure 15. It can be found that the tensile stress-strain of epoxy and polyurethane mainly included two stages: linear elastic stage (ascent section) and fracture failure stage (descent section). Overall, the tensile fracture strain was less than 5% and the brittle fracture was the main failure mode. In contrast, the tensile stress-strain curve of polypropylene mainly included the linear elastic stage, yield stage and plastic deformation stage. Furthermore, the tensile fracture strain was as high as 272%. The main failure mode was the ductile fracture through the plastic deformation, which indicated better resistance to cracking. 

#### 3.6.2. Long Term Performance Analysis 

The long-term properties of three kinds of matrices in the service environment were compared and the predicted retention of tensile strength were presented in Table 8. It can be found that after the service time of three years, the tensile strength retention of polypropylene was still more than 88%. However, the strength retention of epoxy was only about 40%. In addition, the retention of tensile strength for polyurethane was close to 70% when the service time was one year. Therefore, it can be concluded that polypropylene has better corrosive resistance than epoxy and polyurethane, which may obtain great application prospects in bridge engineering. 

#### 3.6.3. Fatigue Performance Analysis

**Figure 16:**
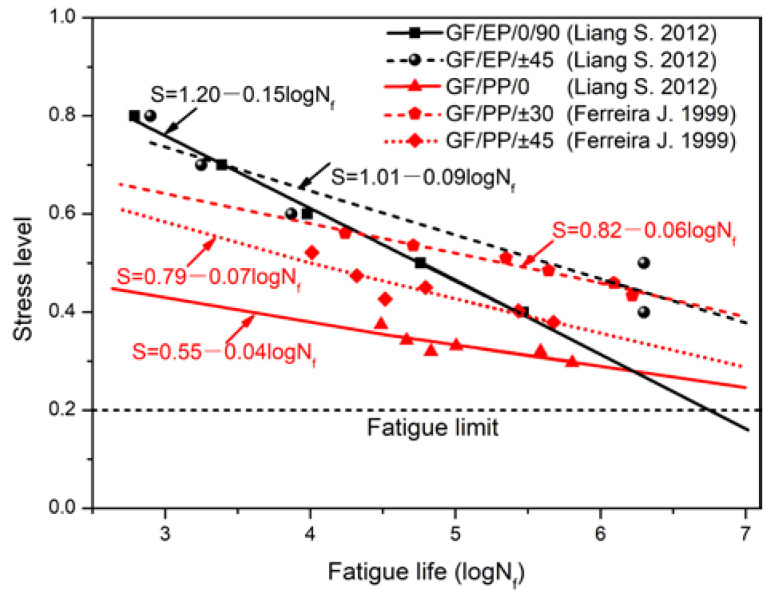
Comparison of fatigue life of glass fiber reinforced epoxy and polypropylene composites. Note, GF, EP and PP are glass fiber, epoxy and polypropylene [69,70].

After analyzing the toughness and long-term property, the fatigue performance analysis was carried out. Figure 16 shows the comparison of fatigue life of glass fiber reinforced epoxy and polypropylene composites, and the fatigue life and degradation rate were shown in Table 9. It can be found that the slope of the S-N curve of glass fiber reinforced epoxy composite was greater than that of polypropylene composites. This indicated that the fatigue life of epoxy composite decreased significantly with the increase in stress levels. Table 9 shows the fatigue life of two kinds of composites at four stress levels. As shown, polypropylene composites had better fatigue resistance, especially for the sheet with a multidirectional layering structure. 

In conclusion, in terms of failure strain, long-term performance and fatigue performance, polypropylene and its composites may achieve great application prospects in engineering applications.

## 4. Conclusions

In the present paper, thermoplastic polypropylene was exposed to the coupling condition of elevated temperature, water immersion and sustained bending loading for as long as 90 days. Water uptake, mechanical and thermal properties tests were conducted to obtain the long-term evolution. The degradation mechanism of mechanical properties was revealed and compared with thermosetting epoxy. Arrhenius model was adopted to obtain the long-term life prediction. Finally, the application prospect analysis of polypropylene in structural engineering was compared with epoxy and polyurethane in terms of failure strain, long-term property and fatigue resistance. The following conclusions can be drawn:

(1) Simplified Fick’s diffusion model can well describe the water absorption and diffusion behavior of two matrices. Water molecules were easier to diffuse in epoxy than polypropylene at the same immersion temperature, which indicated that polypropylene has better hydrophobic behavior compared to epoxy. For example, the ratios of the diffusion coefficient and saturated water uptake between the two matrices were 114.4 and 2.94 at 80 °C.
(2) At the longest immersion time of 90 days, the degradation percentages of tensile strength were 4.7% (40 °C), 7.5% (60 °C) and 8.8% (80 °C), respectively, which had the higher strength retention (>90%). The maximum strength increase multiples of polypropylene/epoxy and polypropylene/polyurethane were 1.95 and 1.75, respectively. The bending loading led to a maximum increase in tensile strength (~1.47%) owing to the oxygen isolation effect.
(3) Tensile strength degradation for polypropylene was the active functional groups reacted with oxygen, resulting in local chain segment fracture. For epoxy and polypropylene, water molecules reacted with the hydroxyl groups or interrupted the intermolecular Van der Waals force/hydrogen bond, resulting in irreversible hydrolysis and property degradation.
(4) Long-term life prediction of tensile strength showed when the service time was 500 days, the retention of tensile strength for polypropylene was close to 90% at 8.8 °C. In contrast, the retention of tensile strength of epoxy was only ~75% for the service time of 120 days. In terms of failure strain, long-term and fatigue performances, polypropylene and its composites have outstanding advantages compared to epoxy and polypropylene, which can achieve great application prospects in engineering applications when considering the complex service environment.

## Figures and Tables

**Table 1:** Exposure conditions of polypropylene and epoxy.

Matrix Type	Exposure Time (Days)	Bending Deflection (mm)/Strain (%)	Exposure Temperature (°C)	Exposure Medium
Polypropylene (PP)	0	20/0.54 (60 °C)	40/60/80	Distilled water
	30			
	60			
	90			
Epoxy (EP)	0	/	40/60/80	Distilled water
	30			
	60			
	90			

**Table 2:** Water absorption fitting parameters of two kinds of matrices immersed at different temperatures.

Matrix Type	Immersion Temperature (°C)	Saturated Water Uptake $M_{\infty }(\%)$	Diffusion Coefficient D (mm^2^/s, ×10^−8^)
Polypropylene	40	0.21	0.93
	60	0.71	4.55
	80	1.02	8.49
Epoxy	40	3.30	41.49
	60	3.16	186.5
	80	3.00	971.0

**Table 3:** Increase ratio of tensile strength retention of polypropylene compared to epoxy and polyurethane immersed in distilled water [51,59].

Immersion Time (days)	40 °C		60 °C	
	PP/EP	PP/PT	PP/EP	PP/PT
30	1.88	1.75	1.38	1.19
90	1.95	1.52	1.27	1.04

**Table 4:** The maximum and ultimate decomposition temperature (°C) of polypropylene and epoxy.

Matrix Type	Control	60 °C-30	40 °C-90	60 °C-90	60 °C-90-FL	80 °C-90
MDT-PP	443.34	444.10	444.52	443.04	452.05	447.30
UDT-PP	455.88	456.10	455.61	454.20	465.99	458.93
MDT-EP	389.76	/	/	391.18	/	/
UDT-EP	410.26	/	/	407.22	/	/

Note, the temperature corresponding to maximum decomposition rate is called as maximum decomposition temperature (MDT), and the inflection point of weight curves is adopted as the ultimate decomposition (UDT). In addition, 40 °C, 60 °C and 80 °C denote the immersion temperature, 30 and 90 denote the immersion time.

**Table 5:** Regression coefficient (*τ*) and degree of fitting (*R*^2^) of life prediction for two kinds of matrices.

Matrix Type	Immersion Temperature/°C	*τ*	*R* ^2^
PP	40	310.2	0.98
	60	224.7	0.97
	80	178.6	0.85
EP [61]	20	374.8	0.99
	40	292.5	0.99
	60	255.7	0.99

**Table 6:** Regression coefficient (*E*_a_/*R*) and degree of fitting (*R*^2^) of life prediction for two kinds of matrices.

Matrix Type	Strength Retention/%	*E*_a_/*R*	*R* ^2^
PP	95	2420	0.91
	90	2420	0.91
	85	2420	0.91
	80	2420	0.91
EP [61]	95	1000	0.99
	85	1000	0.99
	75	1000	0.99
	65	1000	0.99

**Table 7:** Time-shift factor of life prediction of tensile strength for two kinds of matrices.

Matrix Type	Immersion Temperature (°C)	Time-Shift Factor (*TSF*)		
		Shenyang Youth Bridge (*T* = 8.8 °C)	Jiangsu Yangtze River Bridge (*T* = 15.9 °C)	Hainan Century Bridge (*T* = 26.9 °C)
PP	40	2.35	1.91	1.40
	60	3.74	3.03	2.23
	80	5.65	4.58	3.37
EP [61]	20	1.15	1.05	0.92
	40	1.42	1.31	1.15
	60	1.73	1.58	1.39

Note: *T* is the annual mean temperature from China Meteorological Observatory for 2019; *T*_0_ is the immersion temperature in the present paper. The above three typical bridge were selected to simulate the actual bridge service environment.

**Table 8:** Prediction of tensile strength retention for three kinds of matrices (%) [65,67].

Service Time (Years)	PP			EP [67]			PT [65]		
	8.8 °C	15.9 °C	26.9 °C	8.8 °C	15.9 °C	26.9 °C	20 °C-W	20 °C-A	20 °C-S
0.5	95.3	94.5	93.2	68.1	66.2	63.5	57.94 ^a^	57.74 ^a^	57.58 ^b^
1	92.3	91.4	90.2	52.9	51.2	49.0	69.61 ^c^	69.65 ^c^	68.89 ^c^
2	89.6	89.0	88.5	42.8	42.1	41.4	/	/	/
3	88.6	88.4	88.2	40.6	40.4	40.2	/	/	/

Note, ^a^, ^b^ and ^c^ denote the experimental data immersed for 199, 187 and 376 days.

**Table 9:** Comparison to fatigue life (N_f_) and degradation rate of glass fiber reinforced epoxy and polypropylene composites.

Stress Level (%)	GF/EP/0/90 [69]	GF/EP/±45 [69]	GF/PP/0 [70]	GF/PP/±30 [70]	GF/PP/±45 [70]
50	46,416	464,158	18	215,443	13,895
40	215,443	>2,000,000	5623	>2,000,000	372,759
35	464,159	>2,000,000	100,000	>2,000,000	>2,000,000
30	1,000,000	>2,000,000	1,778,279	>2,000,000	>2,000,000
Slope (S-N)	−0.15	−0.09	−0.04	−0.06	−0.07

## Data Availability

Not applicable.
